# Supplementary material for: Expanding Molecular Coverage in Mass Spectrometry Imaging of Microbial Systems Using Metal-Assisted Laser Desorption/Ionization
Source: Microbiol Spectr. 2021 Jul 21;9(1):10.1128/spectrum.00520-21. doi: 10.1128/spectrum.00520-21 (PMC8552643; doi:10.1128/spectrum.00520-21)
Supplement: SUPPLEMENTAL FILE 1 — Supplemental material. Download SPECTRUM00520-21_Supp_1_seq4.docx, DOCX file, 1.6 MB. [file spectrum00520-21_supp_1_seq4.docx]

**Supplemental Information For:**

**­Expanding the molecular coverage in mass spectrometry imaging of microbial systems using metal-assisted laser desorption/ionization**

Jessica K. Lukowski^1^, Arunima Bhattacharjee^1^, Sarah M. Yannarell^2^, Kaitlyn Schwarz^1^, Leslie M. Shor^3^, Elizabeth A. Shank^4^, Christopher R. Anderton^1*^

^11^Environmental Molecular Sciences Division, Pacific Northwest National Laboratory, Richland, WA, USA; ^2^University of North Carolina at Chapel Hill, Chapel Hill, NC, USA; ^3^University of Connecticut, Storrs, CT, USA; ^4^University of Massachusetts Medical School, Worcester, MA, USA

* Christopher.Anderton@pnnl.gov; 902 Battelle Boulevard, Richland, Washington 99352; 509-371-7970

**Table of Contents:**

Figure S1: Workflow of creating microfluidic device……………………………………………..………2

Figure S2: Spectra comparing MetA-LDI-MSI and MALDI-MSI analysis………………..…………...…3

Figure S3: MetA-LDI-MSI of a standard lipid in the microfluidic channel………………………..…...…4

Figure S4: Space charging effects during MetA-LDI-MSI analysis.……………………...………… ……5

Table S1: Number of annotations found in sample preparation optimization……………………………...6

Table S2: Lipid class breakdown of MetA-LDI-MSI and MALDI-MSI analysis……………….………...7

Table S3: Species identified by LESA MS/MS…………………….………………………………...……8

Excel document 1: Complete annotations list from MetA-LDI-MSI and MALDI-MSI

Excel document 2: Fragments of species identified by LESA MS/MS


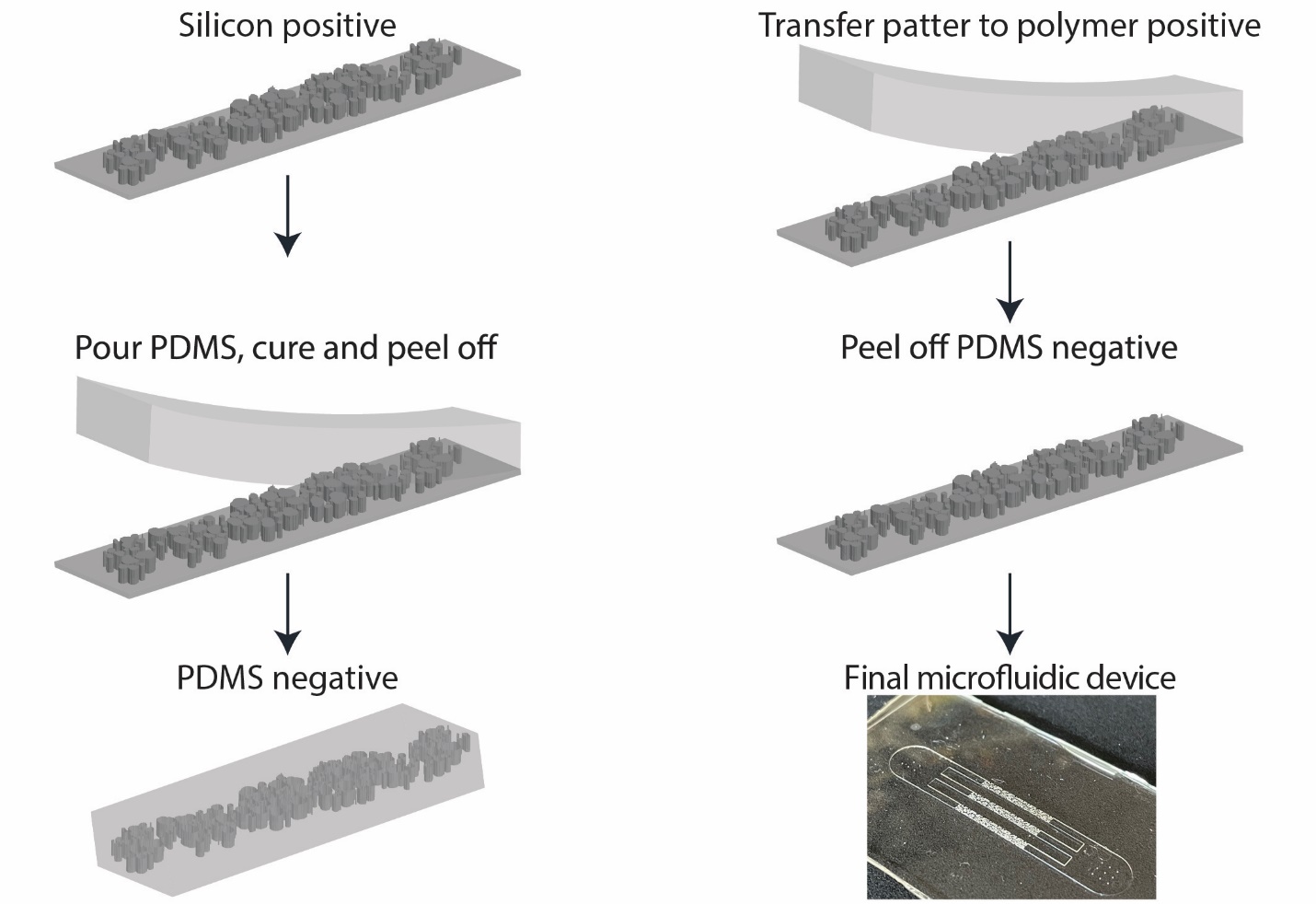


**Supplemental Figure 1.** General workflow followed to create the microfluidic device. The bottom right photograph shows what the device looks like when fabrication is complete.


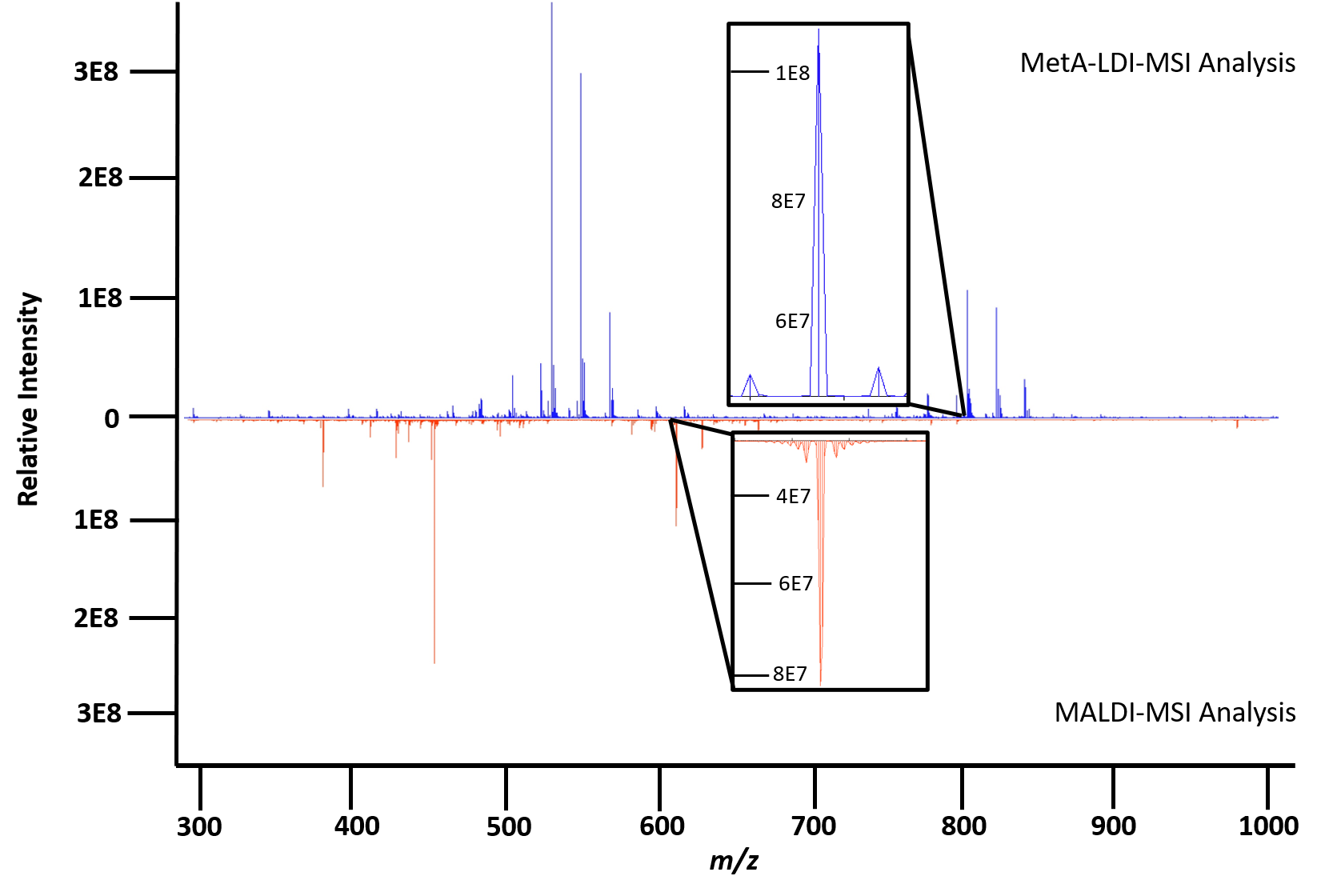


**Supplemental Figure 2.** Representative average spectra of *B. subtilis* colony cut in half and analyzed by both MetA-LDI-MSI (8 nm gold sputter-coated on surface) and MALDI-MSI (10 passes of DHB). Analysis was performed using a 15T LDI-FTICR-MS. Zoomed in peak of Lyso PC (24:0), at *m/z* 608.46552 [M+H]^+^ and *m/z* 804.42425[M+Au]^+^ for MALDI- and MetA-LDI-MSI analysis, respectively, shows MetA-LDI-MSI analysis had a slightly higher relative intensity.


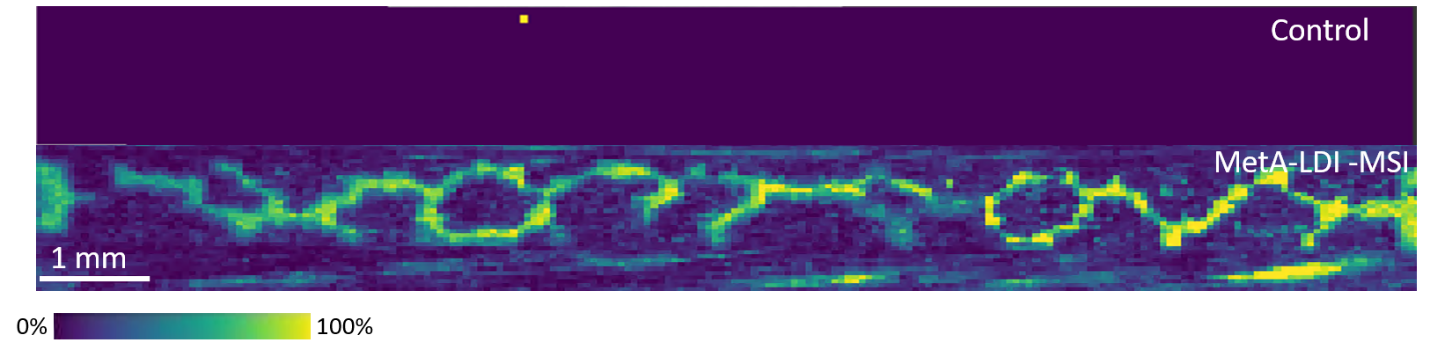


**Supplemental Figure 3.** Standard lipid (1-oleoyl-2-palmitoyl-sn-glycero-3-phosphocholine, *m/z* 760.0834) flowed through the microchannel and analyzed by MetA-LDI-MSI in comparison to a control sample (top image), where no lipid was flowed through the microchannel and no ion signal is visualized.


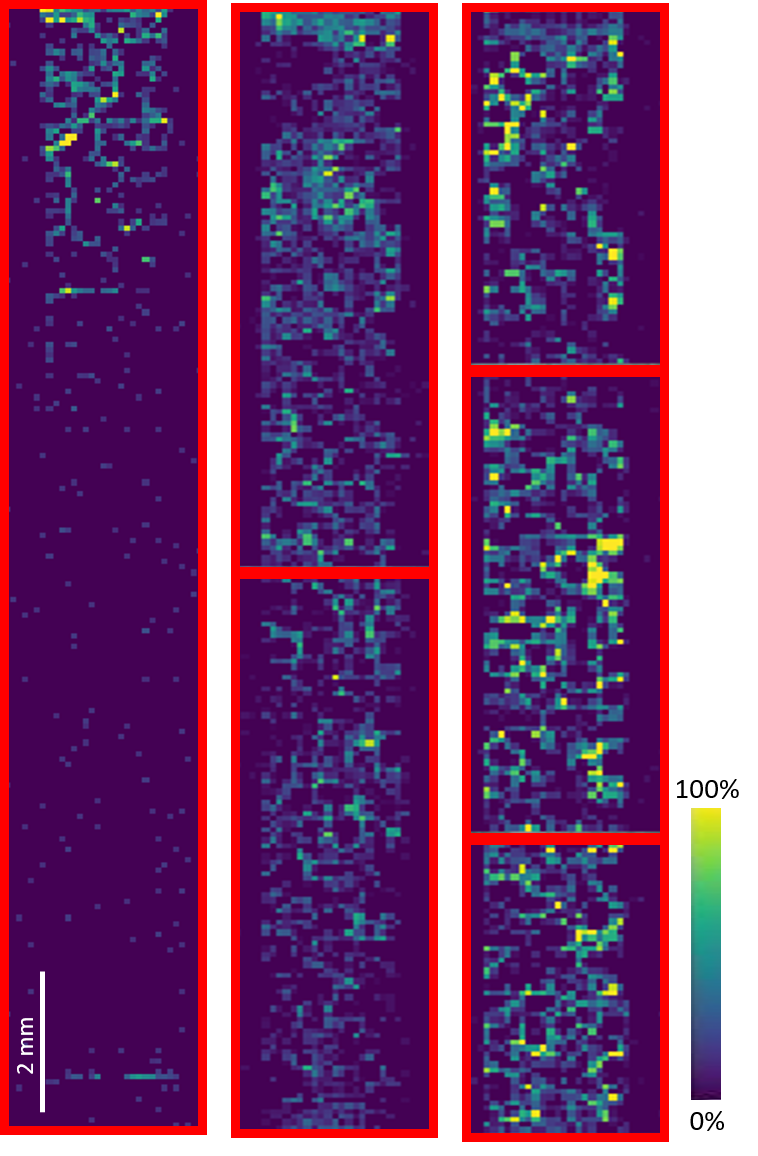


**Supplemental Figure 4.** Space charging effects during analysis of devices with MetA-LDI-MSI analysis. (left) When the microchannel was analyzed as a whole, signal was obtained from the top of the channel, but as the analysis continued signal diminished due to space charging effects. (middle) Breaking the channel up into 2 regions of interest, analyzing the top region first and the bottom region second, helped mitigate some of the space charging effects observed from imaging the whole sample at once. (right) Breaking the microchannel into 3 regions of interest proved to produce the best uniformity of analyte signal, as we analyzed the top of the channel first, followed by the bottom section, and then finally the middle portion. By doing the analysis across multiple regions, separated from one another, it minimized the space charging effects, and it enabled us to image the entire channel uniformly.

**Supplemental Table 1.** Annotations found from *B. subtilis* colonies grown on agar during sample preparation optimization. For MALDI-MSI matrix applications conditions were as follows: DHB (40 mg/mL) in 70% MeOH, 65 °C, 0.1ml/min flow rate, 3 mm track spacing. Annotations were determined through METASPACE (ChEBI, 20%) (1), Metlin (2), and the *B. subtilis* database (3).

|  | MetA-LDI (Au thickness) | | | | | | MALDI sprayer application | | | |
| --- | --- | --- | --- | --- | --- | --- | --- | --- | --- | --- |
|  | 5 nm | 7 nm | 8 nm | 10 nm | 15 nm | 20 nm | 8 passes | 10 passes | 12 passes | 14 passes |
| METASPACE | 37 | 41 | 45 | 43 | 38 | 29 | 29 | 38 | 35 | 19 |
| Metlin | 67 | 75 | 84 | 69 | 62 | 54 | 49 | 53 | 41 | 27 |
| *B. subtilis* database | 94 | 97 | 108 | 95 | 89 | 77 | 91 | 111 | 87 | 64 |
| **TOTAL** | **198** | **213** | **237** | **207** | **189** | **160** | **169** | **202** | **163** | **110** |

**Supplemental Table 2**. Table showing the types of molecular classes detected in MetA-LDI-MSI and MALDI-MSI analysis of a *B. subtilis* colony using the optimal sample preparation conditions per Table S1 (8 nm or 10 passes for MetA-LDI-MSI or MALDI-MSI, respectively). Phosphatidic acid (PA) phosphatidylcholine (PC), phosphatidylethanolamine (PE), phosphatidylglycerol (PG), diacylglycerides (DG), and triacylglycerides (TG) were the main lipid classes in imaging experiments. Number of annotations reflects average between 3 colonies analyzed.

| **Molecular Species** | **Number Detected in MetA-LDI-MSI** | **Number Detected in MALDI-MSI** |
| --- | --- | --- |
| Small Molecules | 36 | 22 |
| PA | 18 | 13 |
| PC | 20 | 30 |
| PE | 21 | 32 |
| PG | 23 | 16 |
| DG | 25 | 9 |
| TG | 28 | 11 |
| Others | 33 | 40 |

**Supplemental Table 3**. Full LESA MS/MS data of *B. subtilis* colony on agar.

| **Molecular Species** | **Parent Ion [M+H]^+^** | **Matching Fragments Identified by MetFrag** | **Detected in MetA- LDI dataset?** | **Detected in MALDI dataset?** |
| --- | --- | --- | --- | --- |
| PG (32:0) | 723.5171 | 10/15 | X | X |
| PG (34:2) | 747.5149 | 10/15 | X | X |
| PA (40:2) | 757.5772 | 12/15 | X |  |
| PC (32:2) | 730.5381 | 11/15 |  | X |
| PC (40:2) | 842.6633 | 11/15 |  | X |
| DG (34:1) | 595.5296 | 11/15 | X |  |
| DG (26:0) | 485.4132 | 11/15 | X |  |
| PE (34:1) | 718.5382 | 9/15 |  | X |
| PE (38:3) | 770.5684 | 14/15 |  | X |
| PE (16:0) | 468.2731 | 13/15 | X | X |
| TG (50:2) | 831.7435 | 8/15 | X |  |
| TG (52:4) | 855.7433 | 9/15 | X |  |

1. Palmer A, Phapale P, Chernyavsky I, Lavigne R, Fay D, Tarasov A, Kovalev V, Fuchser J, Nikolenko S, Pineau C, Becker M, Alexandrov T. 2017. FDR-controlled metabolite annotation for high-resolution imaging mass spectrometry. Nat Methods 14:57–60.

2. Smith CA, O’Maille G, Want EJ, Qin C, Trauger SA, Brandon TR, Custodio DE, Abagyan R, Siuzdak G. 2005. METLIN: a metabolite mass spectral database. Ther Drug Monit 27:747–51.

3. Karp PD, Billington R, Caspi R, Fulcher CA, Latendresse M, Kothari A, Keseler IM, Krummenacker M, Midford PE, Ong Q, Ong WK, Paley SM, Subhraveti P. 2019. The BioCyc collection of microbial genomes and metabolic pathways. Brief Bioinform 20:1085–1093.
